# Social Media and Research Publication Activity During Early Stages of the COVID-19 Pandemic: Longitudinal Trend Analysis

**DOI:** 10.2196/26956

**Published:** 2021-06-17

**Authors:** Sonia L Taneja, Monica Passi, Sumona Bhattacharya, Samuel A Schueler, Sandeep Gurram, Christopher Koh

**Affiliations:** 1 National Institutes of Diabetes and Digestive and Kidney Diseases Digestive Disease Branch Bethesda, MD United States; 2 Urologic Oncology Branch, National Cancer Institute, National Institutes of Health Bethesda, MD United States; 3 Liver Diseases Branch, National Institutes of Diabetes and Digestive and Kidney Diseases Bethesda, MD United States

**Keywords:** coronavirus, COVID-19, social media, gastroenterology, SARS-CoV-2, research, literature, dissemination, Twitter, preprint

## Abstract

**Background:**

The COVID-19 pandemic has highlighted the importance of rapid dissemination of scientific and medical discoveries. Current platforms available for the distribution of scientific and clinical research data and information include preprint repositories and traditional peer-reviewed journals. In recent times, social media has emerged as a helpful platform to share scientific and medical discoveries.

**Objective:**

This study aimed to comparatively analyze activity on social media (specifically, Twitter) and that related to publications in the form of preprint and peer-reviewed journal articles in the context of COVID-19 and gastroenterology during the early stages of the COVID-19 pandemic.

**Methods:**

COVID-19–related data from Twitter (tweets and user data) and articles published in preprint servers (bioRxiv and medRxiv) as well as in the PubMed database were collected and analyzed during the first 6 months of the pandemic, from December 2019 through May 2020. Global and regional geographic and gastrointestinal organ–specific social media trends were compared to preprint and publication activity. Any relationship between Twitter activity and preprint articles published and that between Twitter activity and PubMed articles published overall, by organ system, and by geographic location were identified using Spearman’s rank-order correlation.

**Results:**

Over the 6-month period, 73,079 tweets from 44,609 users, 7164 journal publications, and 4702 preprint publications were retrieved. Twitter activity (ie, number of tweets) peaked in March 2020, whereas preprint and publication activity (ie, number of articles published) peaked in April 2020. Overall, strong correlations were identified between trends in Twitter activity and preprint and publication activity (*P*<.001 for both). COVID-19 data across the three platforms mainly concentrated on pulmonology or critical care, but when analyzing the field of gastroenterology specifically, most tweets pertained to pancreatology, most publications focused on hepatology, and most preprints covered hepatology and luminal gastroenterology. Furthermore, there were significant positive associations between trends in Twitter and publication activity for all gastroenterology topics (luminal gastroenterology: *P*=.009; hepatology and inflammatory bowel disease: *P*=.006; gastrointestinal endoscopy: *P*=.007), except pancreatology (*P*=.20), suggesting that Twitter activity did not correlate with publication activity for this topic. Finally, Twitter activity was the highest in the United States (7331 tweets), whereas PubMed activity was the highest in China (1768 publications).

**Conclusions:**

The COVID-19 pandemic has highlighted the potential of social media as a vehicle for disseminating scientific information during a public health crisis. Sharing and spreading information on COVID-19 in a timely manner during the pandemic has been paramount; this was achieved at a much faster pace on social media, particularly on Twitter. Future investigation could demonstrate how social media can be used to augment and promote scholarly activity, especially as the world begins to increasingly rely on digital or virtual platforms. Scientists and clinicians should consider the use of social media in augmenting public awareness regarding their scholarly pursuits.

## Introduction

COVID-19, caused by the novel coronavirus SARS-CoV-2 (severe acute respiratory syndrome coronavirus), emerged into public view in December 2019 and resulted in a pandemic that has affected six continents, and it continues to indiscriminately affect individuals of all ages, races, and ethnicities. According to the World Health Organization, there have been more than 33,000,000 confirmed cases of COVID-19 globally, including more than 1,000,000 deaths reported by the end of September 2020—only 9 months after its emergence [1]. With time, different countries faced surges in cases straining their health care systems in unprecedented ways. It is during these times that the rapid dissemination of information related to this highly contagious virus and its management has been crucial.

Even though initial experiences related to COVID-19 primarily described respiratory complications, reports of gastrointestinal/gastroenterology (GI) involvement became more evident with increased clinical experience [2]. Although the extent of GI involvement with COVID-19 was uncertain based on early published experiences, it was postulated that this could be substantial due to the identification of the entry mechanism of SARS-CoV-2 that utilizes the angiotensin-2 (ACE2) receptor pathway, which is found throughout the GI tract, liver, and pancreas. Given the pathogen’s similarities to the coronavirus known to cause severe acute respiratory syndrome (SARS) and Middle East Respiratory Syndrome (MERS), investigators suspected that prior experiences with these preceding viruses could provide insight into the current COVID-19 pandemic. Thus, GI luminal manifestations, the involvement of the liver and pancreas, and the management of unique GI patient populations were all considered areas of clinical and research interest [3-5]. Considering the rapid spread of COVID-19 and the consequent interruption of health care services across multiple fronts, the publishing of international experience with COVID-19 along with frequent updates in clinical guidance documents have assisted the GI community in managing this novel disease [6-9]. Furthermore, in an effort to mitigate the spread of infection, endoscopists have encountered significant changes to endoscopic practices by adopting new preprocedure regulations, use of enhanced personal protective equipment, and the rearrangement of endoscopy units to facilitate social distancing [10,11]. Additionally, with the implementation of national “lockdowns,” the ability to share clinical experiences, analyze medical data, and disseminate management strategies for COVID-19 has become reliant on electronic media [12]. During these unprecedented times, the medical community has increasingly utilized social media (eg, Twitter, Facebook, TikTok) for communication and to facilitate interdisciplinary discussion [13,14].

Social media platforms such as Twitter are social networking services through which any user or organization with an account, including those belonging to the scientific and medical communities, can share information and achievements. Compared to other professions, the health care community has been relatively reluctant in utilizing social media for professional purposes related to concerns on its potential impact on employment, medicolegal liability, and relationships among patients and colleagues [15-17]. Nonetheless, as these platforms continue to gain global acceptance and utilization, the ability to collect and analyze data from social media platforms has become essential in understanding health care–related needs, shifting public health interests, and highlighting areas for further medical study [18]. In this study, we aimed to explore COVID-19–related social media activity pertaining to the fields of gastroenterology and hepatology during the initial 6-month period of the COVID-19 pandemic, when knowledge about the virus was new and limited. Furthermore, we aimed to compare activity on social media—specifically Twitter—with that via more traditional channels of medical information sharing and distribution such as publications in medical journals and preprint repositories.

## Methods

### Data Collection for COVID-19–Related Twitter Activity

**Figure 1:**
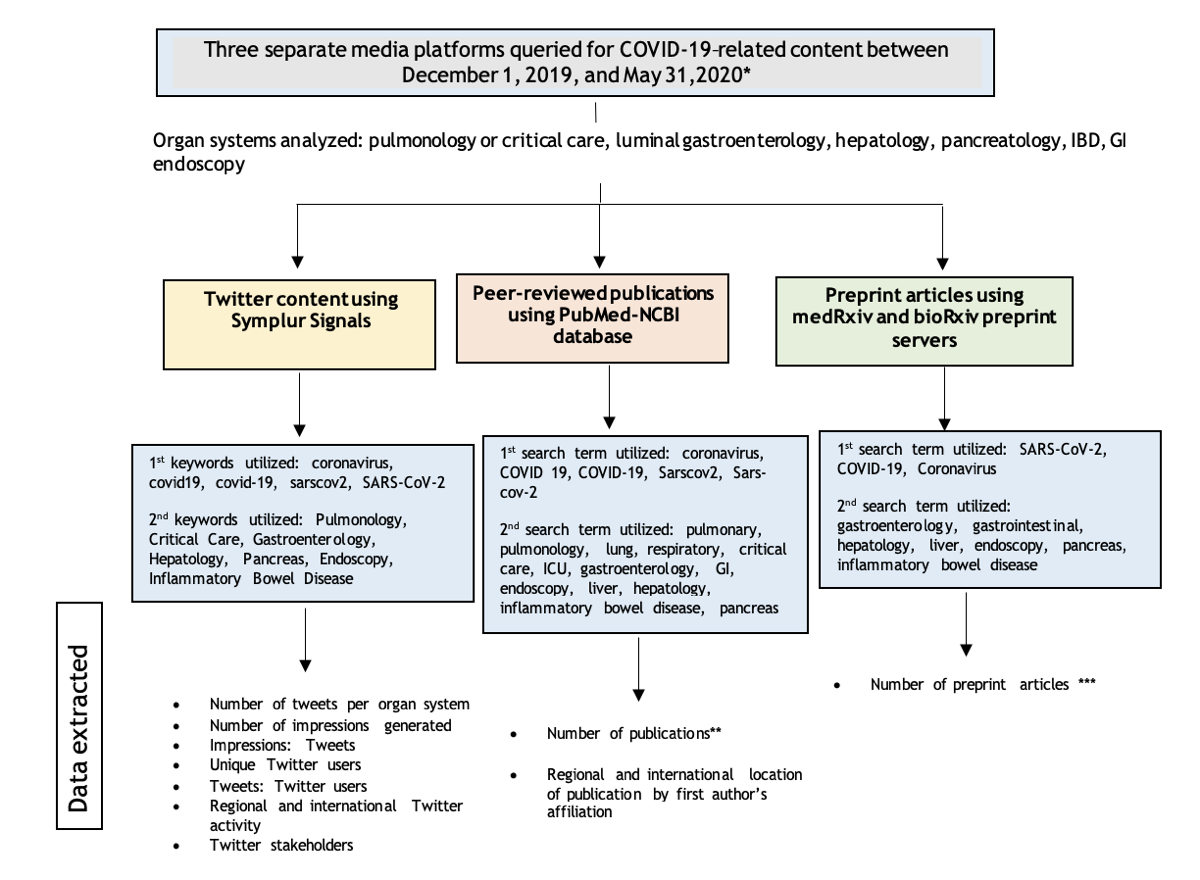
Study flow diagram outlining the data extraction method from 3 media platforms analyzed (Twitter, PubMed-NCBI, and medRxiv or bioRxiv). *Each month of the specific study period was split into half-month intervals for the purposes of analysis. **Duplicate publications from separate searches were individually reviewed and reorganized into the most appropriate subject group in order to eliminate potential for publications to be accounted for more than once. ***A follow-up review of preprint articles pertaining to COVID-19 and gastroenterology that ultimately resulted in formal peer-reviewed journal publications was performed for July 2020. IBD: inflammatory bowel disease; GI: gastrointestinal.

Data were collected using the publicly available Twitter analytics platform Symplur Signals [19]—a health care social media analytics platform that utilizes algorithms with natural language processing to provide in-depth information on Twitter activity. Data on topics related to COVID-19 were collected by performing specific searches categorizing the topics by organ system (Multimedia Appendix 1). Data were captured over the first 6 months of the COVID-19 pandemic, from December 1, 2019, to May 31, 2020. In an effort to capture the longitudinal evolution of social media use during this period, each month was split into half (ie, day 1-15 and day 16 to the end of the month). Data collected included total number of tweets and retweets, total number of impressions, total number of users, and user data, including the place of origin—by country (globally) and by state (within the United States). The ratios of tweets per Twitter user and impressions per tweet were also calculated (Figure 1). Definitions of these terms can be found in Multimedia Appendix 1. 

### Data Collection for COVID-19–Related Preprints and Publications

Preprint articles are research manuscripts shared publicly before peer review, which allows for rapid dissemination of information, thereby helping to inform policy and clinical practice in a timely manner. Preprint repositories have gained considerable attention over the course of the COVID-19 pandemic and have been increasingly utilized for the dissemination of crucial pandemic-related research. For our analysis, preprint articles related to COVID-19 biomedical research were identified using two popular preprint servers: medRxiv and bioRxiv. Specific search terms (see Multimedia Appendix 1) were used to identify and extract COVID-19 preprint articles for each half-month period across the 6-month study period for comparison with Twitter data. Furthermore, a follow-up review of the preprint articles pertaining to COVID-19 and gastroenterology that ultimately resulted in formal peer-reviewed journal publications was performed for the month of July 2020. This was done to account for the delay associated with the formal publication of a preprint article.

For the analysis of peer-reviewed publications, the PubMed–NCBI database was used to search for all publications pertaining to COVID-19 over the 6-month study period. The specific search terms used are detailed in Multimedia Appendix 1. All citations resulting from PubMed searches were recorded, and the search results were further filtered by half-month time intervals, identical to the search methods used for Twitter content and preprint articles for the purposes of comparison. For both preprints and publications, articles were further subgrouped by organ system topic. Duplicate publications from separate searches were individually reviewed and recategorized into the most appropriate subject group, thereby eliminating the potential for publications to be accounted for more than once. Finally, for each publication, the geographic location of the first author’s institution was recorded. 

### Analysis of Social Media, Preprint, and Publication Activity

The primary outcome of the analysis was to identify the peak activity across the 3 platforms, as this shows how efficiently one platform could disseminate information as compared to the others. “Activity” was defined by the number of tweets (via Twitter), or publications (when referring to preprint or publication databases, such as bioRxiv and medRxiv or PubMed, respectively) produced. “Dissemination” could be defined in different ways as referenced by a previous systemic review report published by the Agency for Healthcare Research and Quality (AHRQ); however, for the purpose of this study, it was defined as “the active and targeted distribution of information or interventions via determined channels using planned strategies to a specific public health or clinical practice audience” [20]. In this study, the information distributed is the content of tweets or articles published in preprint repositories or accessible via the PubMed database. Of note, Twitter impressions is a convenient way to measure the exact distribution of the tweet content, as it calculates how many users would have been sent a particular tweet based on the number of followers the user who posted the tweet originally had. Secondary outcomes included (1) peak activity in each platform overall and then by GI subtopic, (2) peak activity in each platform by geographic location, and (3) comparison of trends between the different platforms overall, as well as by GI subtopic (see Multimedia Appendix 1).

Summary statistics of baseline data for tweets, impressions, preprints and PubMed publications are presented as frequencies for categorical data unless otherwise specified. Spearman’s rank-order correlation was performed to determine the relationship between Twitter activity (ie, tweets and impressions) and PubMed publications overall, by organ system and geographic location, as well as by Twitter activity and preprint articles overall and by organ system. Analysis was performed using STATA 15 (StataCorp, LLC) software. Statistical significance was set at *P*<.05. All authors had access to the study data and reviewed and approved the final manuscript.

## Results

### COVID-19–Related Publications and Twitter Activity Trends

**Figure 2:**
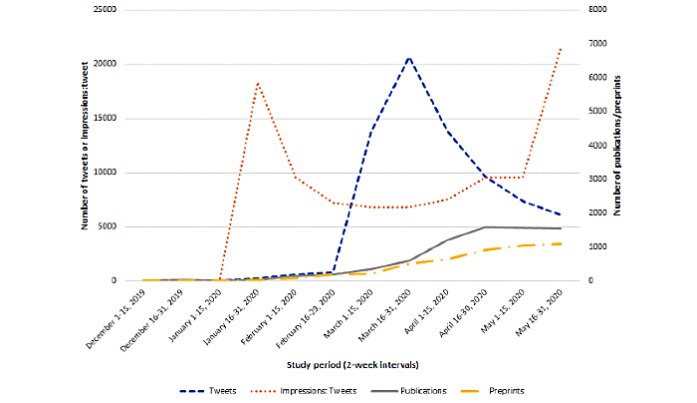
Trend of COVID-19–related tweets, ratio of impressions to tweets, publications and preprints. Twitter activity from December 1, 2019, through May 31, 2020, captured at half-month intervals by using the Twitter analytics platform Symplur Signals. Preprints from medRxiv and bioRxiv repositories were also abstracted during this time period along with publications indexed in the PubMed-NCBI database.

Over the 6-month study period from December 1, 2019, through May 31, 2020, 73,079 tweets were identified from a total of 44,609 users, generating 207,039,610 impressions on the topic of COVID-19. During this same period, 7164 publications pertaining to COVID-19 were found to be indexed in PubMed along with 4702 preprints archived in medRxiv and bioRxiv repositories. The overall summary of Twitter and research publication activity by half-month time interval is shown in Table 1. Twitter activity, with regard to original tweets on the topic of COVID-19 did not appear until the latter half of January 2020, which resulted in 245 original tweets. This activity progressively increased thereafter and peaked during March 16-31, 2020, with 20,660 original tweets before gradually decreasing over the remaining study interval (Figure 2).

**Table 1:** A comparison of productivity trends by half-month intervals of COVID-19–related publication, preprint, and Twitter activity from December 2019 through May 2020.

Informational sources		December 2019			January 2020			February 2020			March 2020			April 2020			May 2020	
		1-15	16-31	1-15		16-31	1-15		16-29	1-15		16-31	1-15		16-30	1-15		16-31
Publications^a^, n		0	0	0		34	135		180	342		588	1196		1586	1561		1541
Preprints^b^, n		0	0	0		35	71		181	217		506	633		922	1051		1086
**Tweets^c^, n**		0	0	0		245	592		815	13797		20660	13845		9636	7399		6090
	Impressions	0	0	0		1,439,197	1,809,224		180,594	30,061,305		44,640,303	33,351,337		29,411,077	22,536,326		41,900,257
	Impressions per tweet	0	0	0		5874	3056		2320	2179		2179	2409		3052	3046		6880
	Unique users	0	0	0		165	362		552	9727		13034	8210		5647	3759		3153
	Tweets per user	0	0	0		1.48	1.64		1.48	1.42		1.59	1.69		1.71	1.97		1.93

^a^Publications indexed in PubMed–NCBI database.

^b^Preprints located in the medRxiv and bioRxiv repositories.

^c^Tweets and associated variables extracted using Symplur Signals search engine.

**Figure 3:**
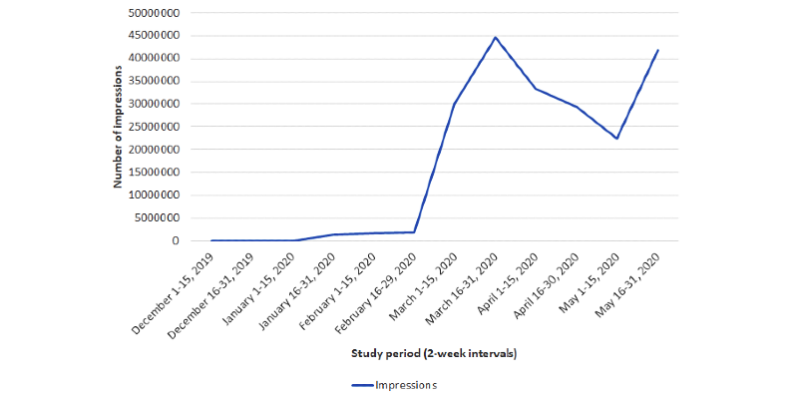
Trend of COVID-19 Twitter impressions. Number of impressions generated from Twitter from December 1, 2019, through May 31, 2020, captured at half-month intervals by using the Twitter analytics platform Symplur Signals.

A similar pattern of activity was observed among the number of Twitter users who posted tweets related to COVID-19, which increased from 165 users to 13,034 users between January and the latter half of March 2020. Twitter impressions followed a similar pattern, with a peak observed during the second half of March 2020. Interestingly, a second peak in impressions was apparent during the latter half of May 2020, which was not observed with regard to the number of tweets and Twitter users (Figure 3). On average, the number of tweets per Twitter user ranged from 1.48 to 1.97. Impressions generated per tweet were initially high (5874 impressions/tweet) in the latter half of January 2020 but did not peak until the latter half of May 2020 (6880 impressions/tweet). Temporal trends are further detailed in Figure 2.

Scientific COVID-19–related articles indexed in PubMed as well as preprints in medRxiv and bioRxiv servers followed a similar trajectory as Twitter activity, with both the first peer-reviewed articles and the first preprints becoming available during the second half of January 2020 [21]. However, unlike Twitter activity that peaked in the second half of March 2020, publications and preprints reached peak activity around the second half of April 2020. Notably, we observed a parallel rise in the number of preprints and PubMed publications (Table 1 and Figure 2).

A moderately strong correlation was demonstrated between Twitter activity (ie, number of tweets) and number of PubMed publications (*ρ*=0.58), as well as between Twitter activity and number of preprints (*ρ*=0.57) across the study duration (*P*<.001 for both; Table 2).

Similarly, there was a moderately strong association between the number of Twitter impressions and PubMed publications (*ρ*=0.56, *P*<.001), as well as between the number of Twitter impressions and preprints (*ρ*=0.54, *P*<.001; Table 3).

**Table 2:** Correlation between Twitter activity (tweets) and PubMed publications and between Twitter activity (tweets) and preprints by organ system. Italicized values indicate statistical significance.

Organ system		Tweets and PubMed publications (*ρ*)	*P* value	Tweets and preprints (*ρ*)	*P* value
Overall trend		0.58	*<.001*	0.57	*<.001*
**Trend by organ system**					
	Pulmonology or critical care	0.8	*.002*	0.8	*.003*
	Luminal gastroenterology	0.7	*.009*	0.6	*.03*
	Hepatology	0.7	*.006*	0.7	*.009*
	Inflammatory bowel disease	0.7	*.006*	0.5	*.07*
	Pancreatology	0.4	.20	0.4	.30
	Gastrointestinal endoscopy	0.7	*.007*	0.7	*.02*

**Table 3:** Correlation between Twitter activity (impressions) and PubMed publications and between Twitter activity (impressions) and preprints by organ system. Italicized values indicate statistical significance.

Organ system		Impressions and PubMed publications (*ρ*)	*P* value	Impressions and preprints (*ρ*)	*P* value
Overall trend		0.56	*<.001*	0.54	*<.001*
**Trend by organ system**					
	Pulmonology or critical care	0.8	*.001*	0.8	*.002*
	Luminal gastroenterology	0.7	*.009*	0.7	*.006*
	Hepatology	0.7	*.005*	0.7	*.006*
	Inflammatory bowel disease	0.8	*.004*	0.4	.20
	Pancreatology	0.5	*.07*	0.3	.30
	Gastrointestinal endoscopy	0.8	*.004*	0.7	*.02*

### COVID-19–Related Twitter, Publication, and Preprint Content Classified by Organ System Topic

#### Overview

**Figure 4:**
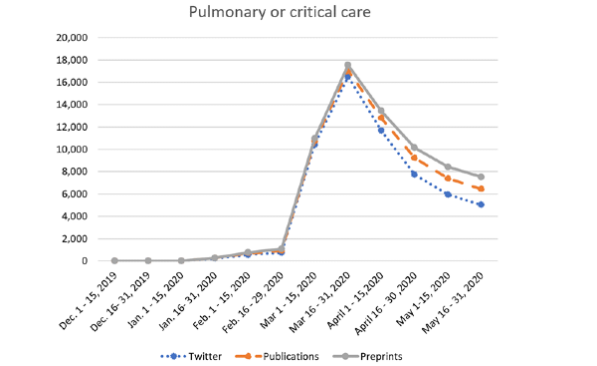
Trend of Twitter, publication, and preprint activity related to COVID-19 and pulmonary or critical care topics. Comparison of the number of tweets posted with articles published in peer-reviewed journals and preprints published pertaining to COVID 19 and pulmonary or critical care at half-month intervals between December 2019 and May 2020.

**Figure 5:**
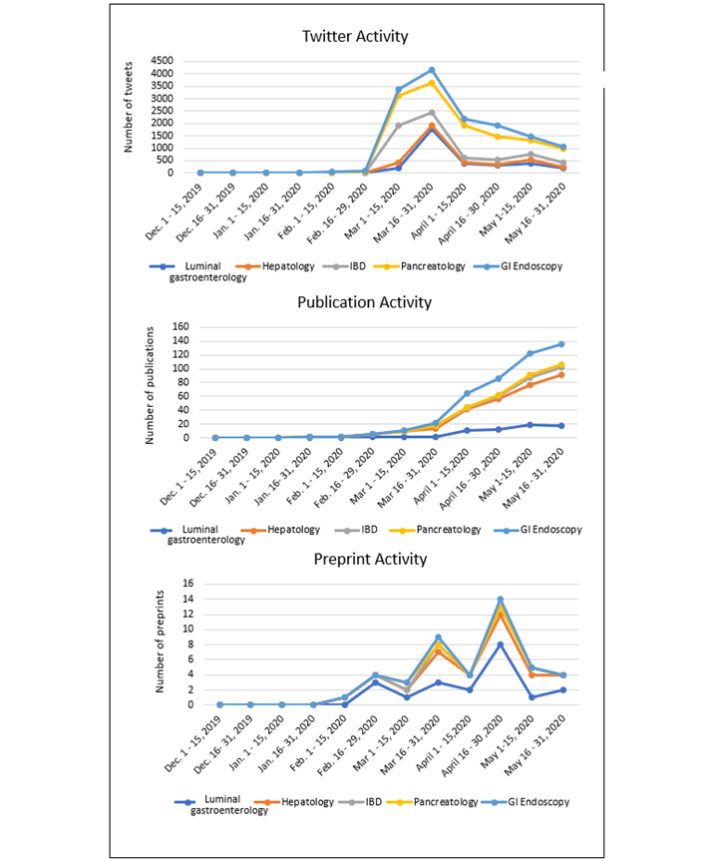
Trend of COVID-19 and gastroenterology subspecialty–related Twitter, publication, and preprint activity. Trend in the number of (A) tweets, (B) publications, and (C) preprints published pertaining to COVID 19 and gastroenterology subspecialty topics at half-month intervals between December 2019 and May 2020. GI: gastrointestinal; IBD: inflammatory bowel disease.

Analysis of Twitter, publication, and preprint data pertaining to the effects of COVID-19 on specific organ system topics are outlined in Table S1 of Multimedia Appendix 2, and trends are illustrated in Figure 4 and Figure 5. The majority of COVID-19–related tweets (58,792/73,079, 80.4%), publications (6713/7164, 93.7%), and preprint articles (4567/4702, 97.1%) covered the topic of pulmonology or critical care. Gastroenterology was a small subset of these topics; however, within the field, the majority of tweets were on the topics of pancreatology (5804/73,079, 7.9%), followed by luminal gastroenterology (3318/73,079, 4.5%), inflammatory bowel disease (2818/73,079, 3.9%), GI endoscopy (1764/73,079, 2.4%), and hepatology (583/73,079, 0.8%). With regard to gastroenterology-related publications, the majority of articles were on the topic of hepatology (236/7250, 3.3%) followed by GI endoscopy (111/7250, 1.5%), luminal gastroenterology (64/7250, 0.9%), IBD (30/7250, 0.4%), and pancreatology (10/7250, 0.1%). Preprint publications were primarily on the topics of luminal gastroenterology (21/4702, 0.4%), hepatology (18/4702, 0.4%), and IBD (3/4702, 0.1%) (see Table S1 in Multimedia Appendix 2).

#### Pulmonology and Critical Care

Approximately 59,000 COVID-19–related tweets analyzed across the study period were on the topic of pulmonology or critical care. The most significant increase in tweets on this topic occurred between February and March 2020, with a nearly 22-fold increase, and an ultimate peak in activity (n=16,489) was observed in the latter half of March (Figure 4 and Table S1 in Multimedia Appendix 2).

A total of 6713 peer-reviewed articles on the topic of pulmonology or critical care and COVID-19 were indexed in PubMed during the 6-month study period, and these first appeared in January 2020. The most significant increase (ie, 2-fold) in the number of publications on COVID-19 and pulmonology or critical care was observed between the latter half of March 2020 and the start of April 2020. As compared to peer-reviewed publications, there were approximately one-third fewer preprint articles (n=4567) in medRxiv and bioRxiv related to COVID-19 and pulmonology or critical care identified during the study period. Moreover, on the topic of pulmonology or critical care and COVID-19, the longitudinal trend in preprint article availability appears to have paralleled publications indexed in PubMed; however, for preprint articles, the most significant rise was observed 2 weeks prior to that observed with PubMed publications, specifically between the first and second half of March (Figure 4 and Table S1 in Multimedia Appendix 2).

There was a strong correlation between both the number of COVID-19–related tweets and peer-reviewed publications (*ρ*=0.8, *P*=.002) as well as between the number of COVID-19–related tweets and preprints (*ρ*=0.8, *P*=.003, respectively; Table 2) on the topic of pulmonology or critical care. Similarly, there was a strong correlation between both pulmonology or critical care–related Twitter impressions and publications (*ρ*=0.8, *P*=.001) as well as between Twitter impressions and preprints (*ρ*=0.8, *P*=.002; Table 3).

#### Gastroenterology

A total of 14,285 tweets concerning the field of gastroenterology and COVID-19 (encompassing subspecialty fields of luminal gastroenterology, IBD, hepatology, GI endoscopy, and pancreatology) were identified during the study period (Figure 5 and Table S1 in Multimedia Appendix 2). Among all tweets recorded during the 6-month study period, 19.6% (14,287/73,079) were on the topic of COVID-19 and gastroenterology. The longitudinal trend in number of gastroenterology-related tweets (including subspecialty gastroenterology fields) paralleled that observed with pulmonology or critical care–related tweets, with an approximate 45-fold increase in the number of tweets spanning the latter half of February 2020 (n=75) and peaking in the second half of March 2020 (n=4171). When further stratified by subspecialty field, the majority of COVID-19 and gastroenterology–related tweets were on the topic of pancreatology (5804/14,287, 40.6%), followed by luminal gastroenterology (3318/14,287, 23.2%), IBD (2647/14,287, 19.7%), GI endoscopy (1764/14,287, 12.3%), and hepatology (583/14,287, 4.1%).

A total of 449 peer-reviewed publications related to COVID-19 and gastroenterology were identified in PubMed during the study period. In contrast to Twitter activity, the majority of these publications were on the topic of hepatology (235/449, 52.3%) followed by GI endoscopy (111/449, 24.6%), luminal gastroenterology (64/449, 14.2%), IBD (30/449, 6.7%), and pancreatology (10/449, 2.2%). Similar to Twitter activity, PubMed publications on the topics of luminal gastroenterology and hepatology first appeared in the latter half of January 2020. The most significant increase in COVID-19 and liver–related publications was observed between the latter half of March (n=12) and early April 2020 (n=31), with an over 2.5-fold increase in the number of publications on this topic. Luminal gastroenterology–related publications, which first appeared in the latter half of February 2020, significantly increased between the second half of March and early April 2020, with a 5-fold increase as detailed in Table S1 in Multimedia Appendix 2.

A total of 45 COVID-19 and gastroenterology–related preprints were archived in medRxiv and bioRxiv servers over the study period. Longitudinal analysis showed that the number of preprints on the topic of gastroenterology peaked in the latter half of April 2020. When further stratified by subspecialty, unlike that observed with peer-reviewed publications, the majority of preprints covered luminal gastroenterology (21/45, 46.7%), followed by hepatology (18/45, 40%), IBD (3/45, 6.7%), GI endoscopy (2/45, 4.4%), and pancreatology (1/45, 2.2%) (Table S1 in Multimedia Appendix 2).

Similar to pulmonology or critical care–related content, there was a strong correlation between tweets and peer-reviewed publications (*ρ*=0.6, *P*=.03) as well as between tweets and preprints (*ρ*=0.7, *P*=.009) on the topic of luminal gastroenterology. Additionally, a strong correlation was identified between both the number of tweets and peer-reviewed publications (*ρ*=0.7, *P*=.006) as well as between tweets and preprints (*ρ*=0.7, *P*=.009) on the topic of COVID-19 and hepatology. A similarly strong correlation was observed between the number of tweets and PubMed publications on the topic of GI endoscopy (*ρ*=0.7, *P*=.007), the number of tweets and preprints on the topic of GI endoscopy (*ρ*=0.7, *P*=.02), and the number of tweets and peer-reviewed publications on the topic of COVID-19 and IBD (*ρ*=0.7, *P*=.008). In contrast, no significant correlation was identified between tweets and peer-reviewed publications or preprints on the topic of COVID-19 and pancreatology (Table 2).

Regarding COVID-19 and luminal gastroenterology content, a strong correlation was observed between both Twitter impressions and peer-reviewed publications (*ρ*=0.7, *P*=.009) as well as between Twitter impressions and preprints (*ρ*=0.7, *P*=.006). Similarly, strong correlations were identified between Twitter impressions and peer-reviewed publications on the topics of COVID-19 and hepatology (*ρ*=0.7, *P*=.005), IBD (*ρ*=0.8, *P*=.004), and GI endoscopy (*ρ*=0.8, *P*=.004). There was no significant correlation between Twitter impressions and publications on the topic of COVID-19 and pancreatology. In evaluating the association between Twitter impressions and preprints, strong associations were found on the topics of hepatology (*ρ*=0.7, *P*=.006) and GI endoscopy (*ρ*=0.7, *P*=.02), whereas no associations were found pertaining to the topics of IBD and pancreatology (Table 3).

### COVID-19 Twitter and Publication Content by Geographic Location

**Figure 6:**
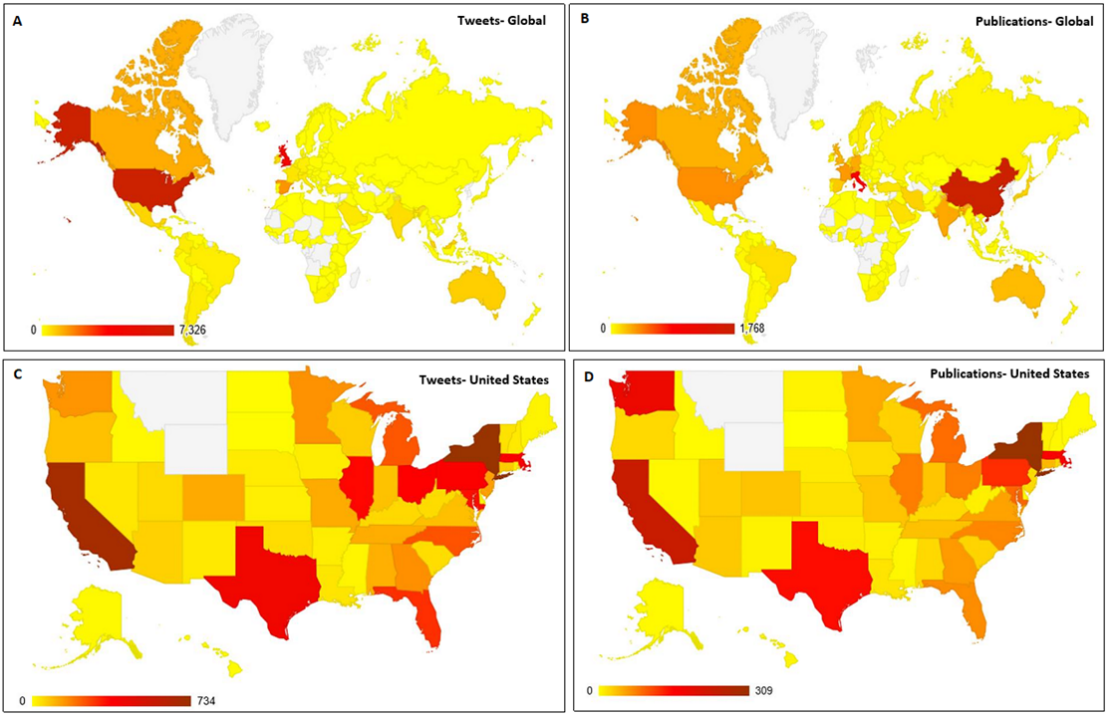
Heat maps illustrating the total number of COVID-19–related tweets (A) across the globe and (C) in the United States, as well as publications indexed in the PubMed-NCBI represented (B) across the globe and (D) in the United States, over the 6-month study period (December 2019 through May 2020). Numbers are represented on the spectrum from the least (yellow) to the highest amount (maroon), as detailed in the legend accompanying each map. Countries or US states shaded white indicate the absence of data for those regions.

The top 5 countries with the highest number of COVID-19–related tweets posted globally over the 6-month study period included the United States (7331/22,215, 33.0%), followed by the United Kingdom (4229/22,215, 19.3%), Spain (1527/22,215, 6.8%), Canada (1174/22,215, 5.3%), and Australia (673/22,215, 3.0%). China generated the highest number of peer-reviewed publications indexed in PubMed (1768/6352, 27.8%) throughout the study period, followed by Italy (915/6352, 14.4%), the United States (389/6352, 6.1%), France (348/6352, 5.5%), and India (303/6352, 4.8%). Figure 6 illustrates the countries with the highest Twitter activity and peer-reviewed publication activity. The top 20 countries with the highest number of tweets and the highest number of peer-reviewed publications are listed in Tables S3 and S4, respectively, in Multimedia Appendix 2. There was a strong correlation between the number of tweets and peer-reviewed publications in both the United States (*ρ*=0.8, *P*=.005) and the United Kingdom (*ρ*=0.8, *P*=.01; see Table S2 in in Multimedia Appendix 2).

Within the United States, when analyzing both Twitter activity and peer-reviewed publications, the state of New York had the highest COVID-19–related activity (11% of tweets and 39.1% of publications) during the study period, followed closely by California (10.3% of tweets and 36.6% of publications). Figure 6 illustrates the Twitter activity and publication activity in the US states. The top 20 US states with the highest number of tweets and peer-reviewed publications are listed in Tables S5 and S6, respectively, in Multimedia Appendix 2.

### COVID-19 Twitter Content by User Stakeholder Designation or Category

Twitter user data entered as the Twitter user’s self-designated health care stakeholder role was analyzed. For the topics of pulmonology or critical care, luminal gastroenterology, hepatology, IBD, and GI endoscopy, the top 2 most active stakeholder categories were doctors/physicians and researchers/academic users. Furthermore, for the topic of pancreatology, advocacy organizations and patient advocates were the most active stakeholder users. The top 15 most active users and their stakeholder roles categorized by Twitter activity for each organ system topic are further detailed in Table S7 in Multimedia Appendix 2.

## Discussion

### Principal Findings

Since its emergence in December 2019, the novel coronavirus SARS-CoV-2 has triggered an unparalleled global response in the fields of science, medicine, public health, and technology. Considering its highly contagious nature, along with the paucity of knowledge and current lack of effective treatment modalities to combat the infection, the need for rapid sharing and dissemination of information has been paramount. In this study, we assessed the dissemination of COVID-19–related information via preprint services, formal peer-reviewed publications, and through the global reach of the social media platform Twitter.

Specifically, we observed that during the second half of March 2020, when COVID-19 was continuing to spread rapidly prompting various nations, including the United States, to enter a state of lockdown, social media activity on Twitter was at its peak, with almost 7000 impressions per tweet analyzed. Furthermore, we observed that Twitter activity was strongly correlated with the published scientific data available to the general public. Although COVID-19 has been predominantly linked with severe pulmonary complications, approximately 20% of the conversations on social media was related to the field of gastroenterology with specific discussions related to hepatology, GI endoscopy, luminal gastroenterology, IBD, and pancreatology. Social media activity was strongly associated with the availability of published data pertaining to all gastroenterology topics with the exception of pancreatology. Finally, in our analysis of data by geographic region of publication, social media activity was most prominent in the regions most affected by the pandemic both globally (in regions where social media via Twitter is not banned) and within the United States, with strong associations between social media and publication data.

In the longitudinal assessment of publication activity, the authors observed that peak social media activity predated peak PubMed publications by approximately 30 days. One possible explanation for this lag interval is the technical delay that typically occurs after acceptance of a peer-reviewed manuscript by a journal and prior to indexing in PubMed. It is worth highlighting that around the peak of PubMed publication activity, we also witnessed a parallel rise in the preprint repository activity. Historically, the use of preprint repositories has allowed researchers to “claim the space” or even to “publish first” in contentious fields of science and research. However, in the face of an evolving pandemic, preprint repositories are serving as a new mode of scientific communication, bypassing the typical lengthy peer-review process and thus allowing for faster dissemination and communication of research and clinical findings related to COVID-19. In fact, in March 2020, when the World Health Organization officially declared COVID-19 a pandemic, 8830 biomedical preprints were published, a 142% increase from the previous year, and medRxiv page views had increased to 15 million a month, as compared to 1 million a month prior to the start of the pandemic [22]. As of July 1, 2020, we were able to document that nearly half of all preprint articles related to COVID-19 and gastroenterology have subsequently made their way into reputable scientific journals, further supporting this theory.

Notably, we observed that physicians, nonmedical doctors, and scientific researchers constituted the lead stakeholder activity for social media use overall during the COVID-19 pandemic. This observation suggests that even during the early stages of the COVID-19 pandemic, social media became an increasingly sought-after tool, likely for the purpose of communicating medical and scientific information. This study confirms—by documenting health care stakeholder activity—that the scientific and medical communities leveraged social media platforms during the early stages of the COVID-19 pandemic more so than other health care stakeholders such as patients or advocacy groups. Previous studies performed during nonpandemic times have demonstrated conflicting evidence regarding the impact of social media on scientific publications related to citation impact, metrics, and viewability [23-26]. This study clearly illustrated that social media activity was greater and also peaked earlier than any of the publication modalities, which is important to consider when investigating how social media could potentially impact research and scientific work in future.

The gastroenterology subtopic of pancreatology is a good example of this potential influence, or rather, the lack thereof. Although pancreatic manifestations (eg, acute pancreatitis) of COVID-19 have been less commonly reported in the literature as compared to other GI symptoms, we unexpectedly observed that Twitter activity on the topic of pancreatology was the highest among all gastroenterology subtopics. However, there was no statistically significant correlation between Twitter and publication activity or between Twitter and preprint activity on the topic of pancreatology, thus implying that the rise of social media activity did not necessarily reflect the same rise in activity in bioRxiv, medRxiv, or PubMed activity. This could possibly be explained by reviewing the stakeholder data for pancreatology-related tweets. For each of the other gastroenterology subfields, stakeholder demographics fell largely under the categories of physicians, nonmedical doctors, or researchers and academic users; however, for the topic of “pancreas,” the most active stakeholders appeared to be advocacy organizations or patient advocates. Upon further review of the top 50 associated hashtags used for the “pancreas” topic, those related to cystic fibrosis support and awareness groups were the most common. Patient advocacy organizations not only play an important part in espousing awareness of specific diseases and patient populations, but they also serve an essential role in the dissemination of information on their behalf, typically in the form or raising awareness on the internet, lobbying directly for change within the government of other institutions, and via marketing and outreach, but likely only indirectly in the context of research activity. Therefore, this increase in Twitter activity may not translate to a proportionate increase in research publication activity on the topic of COVID-19 and pancreatology as demonstrated in this study. Using this topic as an example, further investigation diving deeper into the content of tweets, specific users, and how they translate directly into published work would be helpful to document the direct impact social media has on research activity.

### Limitations

There are several limitations to this study that are worth noting. The first major limitation is that social media platforms, such as Twitter, are not available in several countries, including China. This could help to explain China’s lead in research publication activity as compared with other regions, as publication is likely one of the primary modalities used in this country for disseminating information. Other social media platforms, including WeChat and Sina Weibo, are used in China; however, information regarding the use of these other social media platforms within China is limited to date. Future studies are needed to assess social media activity on these alternative platforms and their association with publications as well as how they compared to the use of Twitter in other nations, such as the United States (where Twitter is not banned). Second, although we were able to account for and reassign duplicate publications for the various categorizations performed, a similar approach could not be guaranteed for Twitter data. The very nature of tweets allows for other users to publish an original tweet to their account generating additional impressions as it is sent to their followers (also known as a “retweet”). Therefore, limiting duplicate tweets may artificially decrease activity. More importantly, certain tweets, or even retweets, may have been assigned to more than one topic area owing to limitations of the Symplur software. Hence, we were unable to limit tweets to a single topic, and as such, this may have artificially boosted the overall number in certain organ system topics, thereby potentially skewing the results of this study.

### Conclusions

In conclusion, this study demonstrates patterns in the utility of social media and publications—both in preprint repositories as well as peer-reviewed journals—for the rapid dissemination of information during a global pandemic of infectious disease. As the world faces this unprecedented public health emergency, this study has reflected on shifting worldwide trends from solely traditional methods of disseminating information (ie, via publications) to more contemporary methods, specifically among the GI community. social media tools like Twitter can be an effective method for educating and informing audiences in real time and via an interactive approach, a feat that cannot always be achieved with more conventional methods (ie, scientific publications). The new media age has resulted in a number of novel avenues for the distribution of information, including Twitter. Utilizing a single modality for dissemination of health care discoveries or information has been shown by the AHRQ to not be as effective as utilizing multiple modalities. It may therefore be time for the medical and scientific communities to cultivate formal social media platforms as effective tools for data sharing and collaboration to augment existing modalities of archival publication.

## Appendix

Multimedia Appendix 1 Search terms used for Twitter, PubMed, and preprint repositories.

Multimedia Appendix 2 Supplementary tables summarizing additional information pertaining to COVID-19–related Twitter, publication, and preprint activity by organ system per half-month time intervals.

## Abbreviations

ACE2: angiotensin-2
AHRQ: Agency for Healthcare Research and Quality
GI: gastrointestinal
IBD: inflammatory bowel disease
MERS: Middle East Respiratory Syndrome
SARS: severe acute respiratory syndrome coronavirus
